# Hierarchical attention and frequency fusion network for endometriotic lesion detection in laparoscopic images

**DOI:** 10.3389/fmed.2026.1875077

**Published:** 2026-07-23

**Authors:** Liusijie Gao, Chunli Sha, Meiling Yang, Yiling Lu, E. Qiukai, Liping Chen

**Affiliations:** 1 Reproductive Medicine Center, Nantong First People's Hospital, Southeast University, Nantong, Jiangsu, China; 2 Department of Obstetrics and Gynecology, Nantong First People's Hospital, Southeast University, Nantong, Jiangsu, China

**Keywords:** computer-aided diagnosis, deep learning, endometriotic lesion detection, frequency domain fusion, hierarchical attention, laparoscopic images, medical image analysis, object detection

## Abstract

Endometriosis is a common gynecological disorder that requires accurate detection of ectopic lesions in laparoscopic images for effective diagnosis and treatment. However, existing deep learning-based detection methods face significant challenges in identifying endometriotic lesions, particularly small lesions that occupy less than 5% of the image area, due to their diverse morphologies, blurred boundaries, and the loss of detail information in deep networks. To address these challenges, we propose HAFF-Net (Hierarchical Attention and Frequency Fusion Network), a novel deep learning framework for endometriotic lesion detection in laparoscopic images. The proposed method integrates two innovative modules into the YOLOv8 backbone: (1) the Hierarchical Lesion Attention Module (HLAM), which simultaneously captures local detail features, global contextual information, and structural characteristics through three complementary branches (Local Attention Branch, Global Attention Branch, and Structural Attention Branch); and (2) the Frequency-Aware Fusion Module (FAFM), which adaptively separates and enhances high-frequency and low-frequency components of feature maps through Fast Fourier Transform, effectively preserving critical detail information for small lesion detection while maintaining overall structural context. On the public GLENDA lesion-detection subset (373 annotated frames from 102 surgical cases), HAFF-Net is compared with plain YOLOv8 and additional detectors under patient-exclusive splits, standard COCO-style metrics, and patient-stratified five-fold cross-validation with bootstrap confidence intervals. Under the standard protocol, five-fold mAP@0.5 reaches 0.291 ± 0.037 for HAFF-Net vs. 0.238 ± 0.038 for YOLOv8n; held-out test recall remains 0.268 (95% CI: 0.184–0.356). We therefore frame the system as preliminary computer-aided decision support for expert review rather than clinically validated autonomous detection; external validation is still required.

## 1 Introduction

Endometriosis is a common gynecological disorder characterized by the abnormal growth of endometrial tissue outside the uterine cavity, such as in the ovaries, fallopian tubes, and pelvic regions. Laparoscopic surgery is currently the gold standard for diagnosing and treating endometriosis, and accurate identification and localization of ectopic lesions in laparoscopic images are crucial for disease diagnosis, staging, and treatment planning. With the rapid development of deep learning techniques, convolutional neural network-based object detection methods have achieved significant progress in medical image analysis, providing new technical approaches for automatic lesion detection in laparoscopic images. However, endometriotic lesions in laparoscopic images often exhibit diverse morphologies, blurred boundaries, and significant size variations. Particularly, small lesions typically occupy less than 5% of the image area, posing substantial challenges for automatic detection systems. Accurate identification and localization of lesions require not only capturing local detail features (such as lesion texture and color variations) but also understanding the global contextual relationships between lesions and surrounding tissues, which imposes higher requirements on the feature extraction and representation capabilities of detection algorithms.

Currently, deep learning-based lesion detection methods for laparoscopic images primarily draw from successful experiences in natural image object detection, such as the YOLO series (1–5) and Faster R-CNN frameworks (6). While these methods perform excellently on natural images, they exhibit significant limitations in endometriotic lesion detection tasks. First, general detection models typically employ single-scale feature extraction strategies, making it difficult to effectively handle the multi-scale characteristics of endometriotic lesions, especially for small lesions (such as superficial lesions), where detail information is easily lost in deep networks (7, 8). Second, existing methods primarily focus on spatial domain feature learning, overlooking the important role of frequency domain information in laparoscopic images. Lesions in laparoscopic images often exhibit unique patterns in the frequency domain, where high-frequency components typically contain detail information such as lesion edges and textures, while low-frequency components carry overall structure and background information, which is crucial for distinguishing lesions from normal tissues (9, 10). Furthermore, traditional attention mechanisms often focus on single-level semantic information, unable to simultaneously capture local details (such as subtle texture changes on lesion surfaces), global context (such as spatial relationships between lesions and surrounding tissues), and structural features (such as lesion boundary shapes), resulting in limited detection accuracy for morphologically complex and boundary-blurred endometriotic lesions (11–13).

Based on the above analysis, endometriotic lesion detection in laparoscopic images faces two key challenges. Challenge 1: Hierarchical representation of lesion features. Endometriotic lesion detection requires simultaneous attention to multiple levels of information: the local level needs to capture subtle textures and color features of lesions (such as red, blue, and black lesion regions with different colors), the global level needs to understand spatial relationships between lesions and surrounding normal tissues (such as peritoneum and ovaries), and the structural level needs to highlight shape and edge information of lesions (such as irregular boundaries and adhesion structures). Existing methods lack effective fusion mechanisms for these multi-level information, making it difficult for models to comprehensively understand the complex features of lesions, especially for morphologically diverse and boundary-blurred superficial lesions. Challenge 2: Effective utilization of frequency domain detail information. Detail information of lesions in laparoscopic images is often hidden in high-frequency components of the frequency domain, while traditional methods only perform feature learning in the spatial domain, unable to fully utilize frequency domain information to enhance lesion detail features. Particularly in small lesion detection (such as lesions with diameters less than 5 mm), the loss of high-frequency detail information severely affects detection accuracy, leading to high false-negative rates.

To address the above challenges, this paper proposes Hierarchical Attention and Frequency Fusion Network (HAFF-Net), a novel laparoscopic image endometriotic lesion detection method that integrates hierarchical lesion attention and frequency-aware fusion. The method is based on the YOLOv8 architecture and introduces two innovative modules in the feature extraction stage. *First, to address Challenge 1, we design a Hierarchical Lesion Attention Module (HLAM)*. This module comprehensively captures endometriotic lesion features by fusing three levels of attention mechanisms: the Local Attention Branch (LAB) employs multi-scale convolutional kernels (3 × 3, 5 × 5, 7 × 7) to extract local detail features at different scales, used for capturing subtle textures and color variations on lesion surfaces; the Global Attention Branch (GAB) captures global contextual information of lesions through global pooling and fully connected layers, used for understanding spatial relationships between lesions and surrounding normal tissues; the Structural Attention Branch (SAB) highlights edge and structural features of lesions through learnable gradient detection operators, used for identifying irregular boundaries and adhesion structures of lesions. The three attention mechanisms are adaptively fused through learnable weights, achieving synergistic enhancement of multi-level lesion features, particularly suitable for morphologically diverse and boundary-blurred endometriotic lesions. *Second, to address Challenge 2, we design a frequency-aware fusion module (FAFM)*. This module performs fast Fourier transform (FFT) on feature maps at the feature level, converting spatial domain features to frequency domain space, then adaptively separates and enhances high-frequency and low-frequency components through the high-frequency enhancement (HFE) and low-frequency preservation (LFP) components. Enhanced high-frequency components are used to highlight detail information of lesions (such as edges, textures, and color boundaries), which is crucial for detecting small lesions and superficial lesions; low-frequency components are used to preserve overall structural information, helping to distinguish lesion regions from normal tissues. Finally, the enhanced frequency domain features are converted back to the spatial domain through inverse fast Fourier transform (IFFT) and fused with original features through residual connections via the adaptive frequency fusion (AFF) component. The two modules are seamlessly integrated into the YOLOv8 backbone network through feature-level hook mechanisms, working collaboratively on multiple feature layers (P3, P4, P5) to jointly improve the model's feature extraction capability and detection accuracy for endometriotic lesions, especially in small lesion detection.

The main contributions of this paper are summarized as follows:

1. We propose *HAFF-Net*, a novel deep learning framework that integrates hierarchical attention and frequency-aware fusion mechanisms for endometriotic lesion detection in laparoscopic images. The proposed architecture effectively addresses the challenges of multi-scale lesion detection and detail preservation in medical imaging.
2. We design a hierarchical attention mechanism that simultaneously captures local detail features, global contextual information, and structural characteristics of lesions through three complementary branches (LAB, GAB, and SAB). This multi-level feature representation is particularly effective for detecting morphologically diverse and boundary-blurred endometriotic lesions.
3. We introduce a frequency domain enhancement approach that adaptively separates and enhances high-frequency and low-frequency components of feature maps. This module effectively preserves critical detail information for small lesion detection while maintaining overall structural context, addressing the challenge of detail loss in deep networks.
4. We evaluate HAFF-Net on GLENDA with patient-exclusive splits, plain YOLOv8 and stronger detection baselines under identical training, patient-stratified cross-validation with uncertainty estimates, and module ablations (HLAM/FAFM), while explicitly discussing dataset size limits and the lack of external validation.

## 2 Related work

### 2.1 Medical image analysis and computer-aided diagnosis

The field of medical image analysis and computer-aided diagnosis (CAD) has seen significant advancements aimed at improving disease detection, diagnosis, and treatment planning. Early efforts, such as those by Kallergi et al. (14), focused on developing algorithms for mammographic microcalcification clusters, emphasizing automated detection, segmentation, and classification techniques utilizing wavelet filters and artificial neural networks. These foundational approaches laid the groundwork for more sophisticated methods in CAD systems.

In the context of breast cancer detection, Giger (15) provided a comprehensive review of CAD applications across various imaging modalities, including mammography, ultrasound, and MRI. The study highlighted how computer-generated outputs assist radiologists in detection and diagnosis, ultimately enhancing interpretation accuracy and patient care. This multi-modality approach underscores the importance of integrating diverse imaging techniques within CAD frameworks.

Recent developments have incorporated advanced segmentation and shape analysis methods. Suri et al. (16) discussed innovations such as the level set method and shape-from-shading models, which contribute to more precise segmentation of medical images. These techniques are crucial for delineating anatomical structures and pathological regions, facilitating accurate diagnosis.

The challenge of limited labeled data in medical imaging has prompted the adoption of weak supervision strategies. Kandemir and Hamprecht (17) evaluated multiple instance learning frameworks, demonstrating their effectiveness in CAD applications like Barrett's cancer diagnosis and diabetic retinopathy screening. These methods enable models to learn from weakly labeled datasets, improving robustness and applicability.

Classification techniques have also evolved, with (18) reviewing state-of-the-art image classification methods for disease diagnosis. Their survey emphasizes the integration of biomedical records with imaging data, highlighting the importance of combining multiple data sources for comprehensive analysis.

The advent of deep learning has revolutionized medical image analysis. Shen et al. (19) introduced fundamental deep learning techniques and their successful applications in registration, detection, segmentation, and disease prognosis. Similarly, Litjens et al. (20) provided an overview of convolutional neural networks (CNNs) in CAD, emphasizing their role in enhancing diagnostic efficiency over the past two decades. Yu et al. (21) further surveyed CNN applications, noting significant progress and improvements in accuracy and robustness.

In particular, 3D deep learning approaches have gained prominence for their ability to handle volumetric data. Niyas et al. (22) reviewed 3D convolutional neural networks for medical image segmentation, highlighting their potential in clinical decision-making and treatment planning. The integration of deep neural networks with transfer learning has also shown promising results in specific disease classification tasks. Aljuaid et al. (23) demonstrated this by achieving high accuracy in breast cancer classification using deep neural networks such as ResNet, Inception-V3, and ShuffleNet, trained on publicly available datasets.

Overall, the literature indicates a clear trajectory toward increasingly sophisticated, accurate, and automated CAD systems. The integration of deep learning techniques, multi-modality imaging, and innovative segmentation methods continues to push the boundaries of medical image analysis, promising improved diagnostic capabilities and patient outcomes.

### 2.2 Endometriosis detection and classification

The detection and classification of endometriosis encompass a range of diagnostic modalities, each with varying degrees of accuracy and clinical utility. Imaging techniques such as magnetic resonance imaging (MRI) and ultrasonography have been extensively studied for their non-invasive capabilities. Kim et al. (24) demonstrated that MRI is effective in detecting rectal endometriosis, although it shows limitations in identifying submucosal or mucosal involvement compared to rectal endoscopic sonography (RES). This suggests that while MRI can be valuable for certain lesion localizations, its accuracy may be constrained in more superficial tissue layers.

Ultrasound-based methods, particularly transvaginal sonography (TVS), have also been evaluated for their diagnostic performance. Montanari et al. (25) conducted a prospective multicenter study involving 745 women, revealing that sonography can accurately detect ovarian and deep endometriosis when assessed using the #Enzian classification. Keckstein et al. (26) further emphasized the clinical relevance of TVS, highlighting its utility in pre-surgical staging and classification, which can inform surgical planning and risk assessment.

In addition to imaging, surgical inspection remains the gold standard for endometriosis detection, with (27) advocating for a comprehensive “toolbox approach” that integrates multiple diagnostic and classification systems. This approach aims to maximize diagnostic accuracy and facilitate better patient management, emphasizing the importance of histological confirmation during laparoscopy.

Emerging diagnostic technologies are also being explored. Pal et al. (28) introduced an impedimetric immunosensor utilizing electrochemical impedance spectroscopy (EIS) to detect A1BG, a potential biomarker for endometriosis. The implementation of machine learning-based classification models with this sensor indicates promising avenues for precise, minimally invasive diagnosis.

Recent advances in computational methods have further enhanced diagnostic capabilities. Butler et al. (29) investigated the application of self-supervised pre-training techniques on multi-modal imaging data, demonstrating their effectiveness in improving classification performance. These methods address challenges related to data scarcity and modality variability, thereby enhancing the generalizability of endometriosis detection models.

Moreover, Zhang et al. (30) proposed a knowledge distillation framework to improve the detection of posterior deep endometriosis (POD) obliteration from MRI by leveraging unpaired TVUS data. This approach underscores the potential of combining different imaging modalities and machine learning strategies to overcome modality-specific limitations.

Finally, innovative bioelectronic sensing approaches are being explored for systemic detection. Sanchez et al. (31) developed an insect brain-inspired bioelectronic sensor capable of differentiating endometriotic from endometrial models based on emitted volatile organic compounds (VOCs), achieving an accuracy of 89%. This novel method highlights the potential for non-invasive, rapid detection systems that could complement existing diagnostic tools.

In summary, the current landscape of endometriosis detection and classification is characterized by a combination of traditional imaging, surgical confirmation, and emerging bioelectronic and machine learning-based technologies. The integration of these modalities, along with advancements in computational techniques, holds promise for more accurate, non-invasive, and early diagnosis of endometriosis.

### 2.3 Deep learning on laparoscopic endometriosis (GLENDA and related work)

Recent studies directly target endometriosis in laparoscopic imagery. Bondarenko et al. (32) compared Faster R-CNN and YOLOv9 on a large custom video-derived detection dataset and emphasized that stratified, leakage-aware training is critical when frames originate from the same procedure; they noted that GLENDA remains valuable but limited in annotated frame count and lesion diversity. Zhu et al. (33) evaluated explainable classifiers (ResNet50, EfficientNet-B2, EdgeNeXt_Small, ViT-Small/16) on GLENDA using patient-aware five-fold cross-validation and Grad-CAM/SHAP analysis for binary pathology classification. Our work differs by addressing *lesion-level bounding-box detection* with architectural modules tailored to small, low-contrast implants (HLAM and FAFM), while adopting the same methodological lessons regarding patient-level partitioning and reporting uncertainty rather than single-split point estimates.

## 3 Method

**Figure 1:**
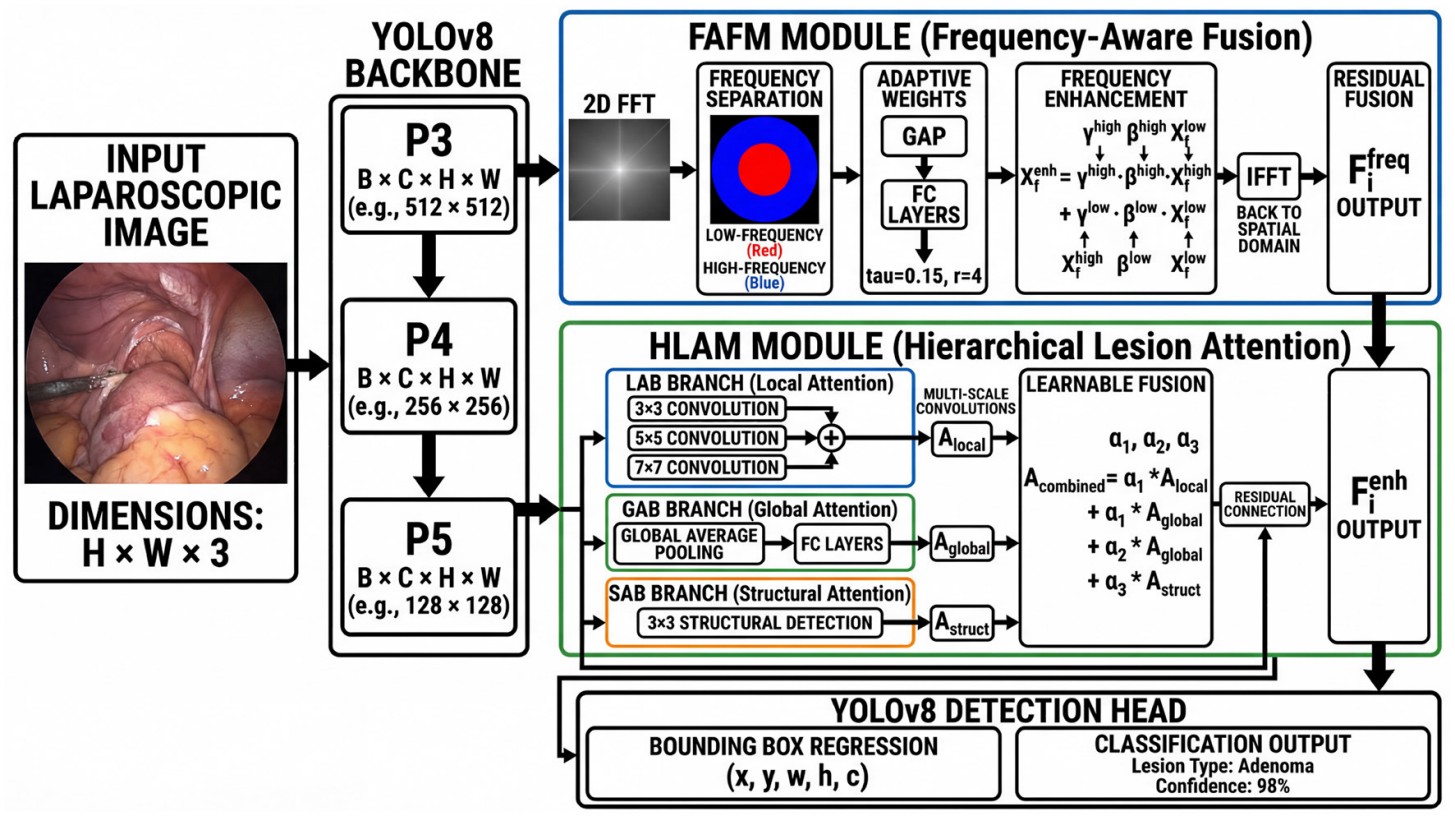
Detailed architecture of the proposed HAFF-Net.

This section presents the detailed architecture of the proposed HAFF-Net framework for endometriotic lesion detection in laparoscopic images, as shown in Figure 1. Compared with natural-image object detection, laparoscopic imagery exhibits pronounced specular highlights, blood-induced chromatic deviations, smoke blur, and severe class imbalance caused by small and shallow lesions (often <5% of the image area). These medical imaging characteristics motivate the design of modules that emphasize fine-grained lesion morphology while preserving the global uterine context. We first introduce the overall architecture, then detail the hierarchical lesion attention module (HLAM) and the frequency-aware fusion module (FAFM). The section concludes with the training workflow, loss design, and complexity analysis, accompanied by a glossary that clarifies the mathematical symbols used throughout the section.

### 3.1 Overall architecture

The proposed HAFF-Net is built upon the YOLOv8 backbone, which serves as the feature extraction network. HAFF-Net integrates two innovative modules into the backbone network at multiple feature layers (P3, P4, P5). Given an input laparoscopic image **I**∈ℝ^*H*×*W*×3^, the YOLOv8 backbone extracts multi-scale features ${\left\{{\text{F}}_{i}\right\}}_{i=3}^{5}$, where ${\text{F}}_{i}\in {\mathbb{R}}^{B\times C_{i}\times H_{i}\times W_{i}}$ represents the feature map at layer *i*, with *B* being the batch size, *C*_*i*_ the number of channels, and *H*_*i*_×*W*_*i*_ the spatial dimensions.

At each feature layer, the feature map **F**_*i*_ is first processed by the FAFM module to suppress smoke-/blood-induced low-contrast artifacts and emphasize fine structural cues (e.g., irregular lesion edges), producing ${\text{F}}_{i}^{freq}$. Subsequently, the HLAM module is applied to ${\text{F}}_{i}^{freq}$ to capture lesion-specific saliency patterns that reflect the heterogeneous colors and textures encountered during laparoscopic inspection, yielding the final enhanced feature ${\text{F}}_{i}^{enh}$ (Equations 1–26):


$$\begin{array}{l} {\text{F}}_{i}^{enh}=\text{HLAM}\left(\text{FAFM}\left({\text{F}}_{i}\right)\right) \end{array}$$ (1)

The enhanced features are then fed into the detection head for bounding box regression and classification.

### 3.2 Hierarchical lesion attention module (HLAM)

The HLAM module captures multi-level lesion features through three complementary branches that address distinct imaging challenges: (i) small, punctate red/black lesions (LAB), (ii) contextual relationships with surrounding organs such as peritoneum or ovaries (GAB), and (iii) adhesion-induced structural boundaries (SAB). Given an input feature map **X**∈ℝ^*B*×*C*×*H*×*W*^, the HLAM module generates an enhanced feature map through hierarchical attention mechanisms tailored to these pathological characteristics.

#### 3.2.1 Local attention branch (LAB)

The LAB employs multi-scale convolutional kernels to capture local detail features at different scales. This design reflects the clinical observation that endometriotic lesions vary from pinpoint superficial spots to irregular plaque-like regions. For a set of kernel sizes $K=\left\{k_{1},k_{2},k_{3}\right\}=\left\{3,5,7\right\}$, the local attention at scale *k*_*j*_ is computed as:


$$\begin{array}{l} {\text{A}}_{j}^{local}=\sigma \left({\text{Conv}}_{1\times 1}\left(\text{ReLU}\left(\text{BN}\left({\text{Conv}}_{k_{j}\times k_{j}}^{group}\left(\text{X}\right)\right)\right)\right)\right) \end{array}$$ (2)

where ${\text{Conv}}_{k_{j}\times k_{j}}^{group}$ denotes a grouped convolution with kernel size *k*_*j*_, BN is batch normalization, ReLU is the rectified linear unit activation, and σ is the sigmoid function. The grouped design reduces computation while allowing channel-specialized filters to focus on specific chromatic and textural patterns (e.g., blue-purple vs. red lesions). The multi-scale local features are then concatenated and fused:


$$\begin{array}{l} {\text{A}}^{local}=\sigma \left({\text{Conv}}_{1\times 1}\left(\left[{\text{A}}_{1}^{local};{\text{A}}_{2}^{local};{\text{A}}_{3}^{local}\right]\right)\right) \end{array}$$ (3)

where [·;·;·] denotes channel-wise concatenation.

#### 3.2.2 Global attention branch (GAB)

The GAB captures global contextual information through global average pooling followed by fully connected layers. This process mimics the diagnostic practice in laparoscopy where surgeons evaluate lesion distribution relative to uterine landmarks:


$$\begin{array}{l} {\text{A}}^{global}=\sigma \left({\text{W}}_{2}\cdot \text{ReLU}\left({\text{W}}_{1}\cdot \text{GAP}\left(\text{X}\right)\right)\right) \end{array}$$ (4)

where $\text{GAP}\left(\text{X}\right)=\frac{1}{HW}\overset{H}{\underset{h=1}{\sum }}\overset{W}{\underset{w=1}{\sum }}{\text{X}}_{:,:,h,w}$ is the global average pooling operation, ${\text{W}}_{1}\in {\mathbb{R}}^{C/r\times C}$ and ${\text{W}}_{2}\in {\mathbb{R}}^{C\times C/r}$ are learnable weight matrices with reduction ratio *r* = 4, and the result is reshaped to ℝ^*B*×*C*×1 × 1^. The resulting attention highlights lesion candidates that conform to anatomically plausible regions (e.g., uterosacral ligaments, ovarian surface).

#### 3.2.3 Structural attention branch (SAB)

The SAB highlights structural features such as edges and fibrotic adhesions through learnable gradient detection operators. This branch is particularly useful for delineating stellate scar tissue or peritoneal puckering that frequently accompanies deep infiltrating lesions:


$$\begin{array}{l} {\text{A}}^{struct}=\sigma \left({\text{Conv}}_{1\times 1}\left(\text{ReLU}\left(\text{BN}\left({\text{Conv}}_{3\times 3}\left(\text{X}\right)\right)\right)\right)\right) \end{array}$$ (5)

#### 3.2.4 Attention fusion

The three attention maps are adaptively fused using learnable weights **α** = [α_1_, α_2_, α_3_]:


$$\begin{array}{l} \alpha =\text{softmax}\left(\alpha_{0}\right) \end{array}$$ (6)

$$\begin{array}{l} {\text{A}}^{combined}=\alpha_{1}{\text{A}}^{local}+\alpha_{2}{\text{A}}^{global}+\alpha_{3}{\text{A}}^{struct} \end{array}$$ (7)

where **α**_0_ is the initial learnable parameter vector. The learnable fusion allows the network to prioritize local, global, or structural cues according to the patient's lesion phenotype. The combined attention is then applied to the input feature map with residual connection:


$$\begin{array}{l} {\text{X}}^{enh}={\text{Conv}}_{3\times 3}\left(\text{X}⊙{\text{A}}^{combined}+\text{X}\right) \end{array}$$ (8)

where ⊙ denotes element-wise multiplication.

### 3.3 Frequency-aware fusion module (FAFM)

The FAFM module enhances feature representations by exploiting frequency domain information. In laparoscopic videos, smoke, specular highlights, and motion blur diminish high-frequency lesion edges, whereas illumination fall-off affects low-frequency structures. Given an input feature map **X**∈ℝ^*B*×*C*×*H*×*W*^, the module performs frequency domain analysis and adaptive fusion to restore diagnostically important frequencies.

#### 3.3.1 Frequency domain transformation

First, the input feature map is transformed to the frequency domain using a 2D fast Fourier transform (FFT), converting spatial patterns into spectra that separate structural and textural cues:


$$\begin{array}{l} {\text{X}}_{f}=F\left(\text{X}\right)=\text{FFT2}\left(\text{X}+j\cdot \text{0}\right) \end{array}$$ (9)

where ${\text{X}}_{f}\in {\mathbb{C}}^{B\times C\times H\times W}$ is the frequency domain representation, and *j* is the imaginary unit. The frequency spectrum is then shifted to center the low-frequency components:


$$\begin{array}{l} {\text{X}}_{f}^{shift}=\text{fftshift}\left({\text{X}}_{f}\right) \end{array}$$ (10)

#### 3.3.2 Frequency component separation

The frequency spectrum is separated into high-frequency and low-frequency components using distance-based masks. This separability enables selective boosting of lesion edges (high-frequency) while preserving overall organ topology (low-frequency). For each spatial location (*h, w*), the distance from the center (*H*/2, *W*/2) is computed:


$$\begin{array}{l} d\left(h,w\right)=\sqrt{{\left(h-H/2\right)}^{2}+{\left(w-W/2\right)}^{2}} \end{array}$$ (11)

The normalized distance is:


$$\begin{array}{l} \bar{d}\left(h,w\right)=\frac{d\left(h,w\right)}{\sqrt{{\left(H/2\right)}^{2}+{\left(W/2\right)}^{2}}} \end{array}$$ (12)

The frequency masks are defined as:


$$M^{low}(h,w)=\{\begin{array}{ll} 1 & \text{if }\bar{d}(h,w)<\tau \\ 0 & \text{otherwise} \end{array},\;M^{high}=1-M^{low}$$ (13)

where τ = 0.15 is the cutoff ratio empirically chosen to retain the central 30% of the spectrum as structural information. The frequency components are separated as:


$$\begin{array}{l} {\text{X}}_{f}^{low}={\text{X}}_{f}^{shift}⊙{\text{M}}^{low},\;{\text{X}}_{f}^{high}={\text{X}}_{f}^{shift}⊙{\text{M}}^{high} \end{array}$$ (14)

#### 3.3.3 Adaptive frequency enhancement

Adaptive weights are learned from the input feature map to balance high-frequency and low-frequency components. Clinically, this allows the network to adjust its sensitivity when encountering specular spots or hemorrhagic tissues:


$$\begin{array}{l} \left[\beta^{high},\beta^{low}\right]=\text{softmax}\left(\text{Conv}\left(\text{GAP}\left(\text{X}\right)\right)\right) \end{array}$$ (15)

The frequency components are enhanced using learnable parameters **γ** = [γ^*high*^, γ^*low*^] that scale the adaptive weights. γ^*high*^>1 boosts subtle lesion edges, whereas γ^*low*^ stabilizes global appearance cues such as uterine contour:


$$\begin{array}{l} {\text{X}}_{f}^{enh}=\gamma^{high}\beta^{high}{\text{X}}_{f}^{high}+\gamma^{low}\beta^{low}{\text{X}}_{f}^{low} \end{array}$$ (16)

#### 3.3.4 Inverse transformation and fusion

The enhanced frequency domain features are transformed back to the spatial domain:


$$\begin{array}{l} {\text{X}}^{freq}=\text{Re}\left(\text{IFFT2}\left(\text{ifftshift}\left({\text{X}}_{f}^{enh}\right)\right)\right) \end{array}$$ (17)

where Re(·) extracts the real part. Finally, the frequency-enhanced features are fused with the original features through residual connections to preserve baseline predictions while introducing frequency corrections:


$$\begin{array}{l} {\text{X}}^{enh}={\text{Conv}}_{3\times 3}\left({\text{Conv}}_{1\times 1}\left({\text{X}}^{freq}\right)\right)+\text{X} \end{array}$$ (18)

### 3.4 Training procedure

The training procedure of HAFF-Net is summarized in Algorithm 1 and Table 1. The model is trained end-to-end using a combination of detection loss and auxiliary losses. We adopt batch size *B* and Adam optimizer with cosine-annealed learning rate. The overall loss function is:


$$\begin{array}{l} L_{total}=L_{det}+\lambda_{1}L_{att}+\lambda_{2}L_{freq} \end{array}$$ (19)

Algorithm 1 Training procedure of HAFF-Net. 
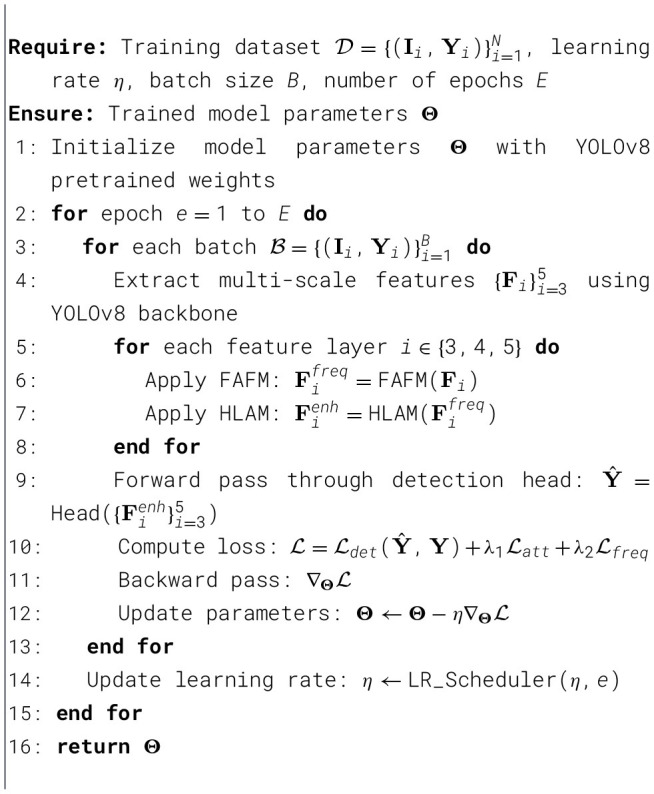

**Table 1:** Stepwise training workflow of HAFF-Net.

Step	Description
1. Initialization	Load YOLOv8 pretrained weights. Initialize HLAM and FAFM modules with Xavier uniform initialization.
2. Feature extraction	Forward pass input image **I** through YOLOv8 backbone to obtain multi-scale features {**F**_3_, **F**_4_, **F**_5_}.
3. Frequency enhancement	For each feature layer *i*∈{3, 4, 5}: Apply FAFM to enhance frequency domain information: ${\text{F}}_{i}^{freq}=\text{FAFM}\left({\text{F}}_{i}\right)$.
4. Attention enhancement	For each feature layer *i*∈{3, 4, 5}: Apply HLAM to generate hierarchical attention: ${\text{F}}_{i}^{enh}=\text{HLAM}\left({\text{F}}_{i}^{freq}\right)$.
5. Detection	Forward pass enhanced features ${\left\{{\text{F}}_{i}^{enh}\right\}}_{i=3}^{5}$ through detection head to obtain predictions $\hat{\text{Y}}$.
6. Loss computation	Compute total loss: $L=L_{det}+\lambda_{1}L_{att}+\lambda_{2}L_{freq}$, where λ_1_ = 0.1, λ_2_ = 0.1.
7. Backward pass	Compute gradients $\nabla_{\Theta }L$ using backpropagation to incorporate both detection and frequency/attention regularization signals.
8. Parameter update	Update model parameters using Adam optimizer: $\Theta \leftarrow \Theta -\eta \nabla_{\Theta }L$.
9. Learning rate schedule	Update learning rate using cosine annealing: $\eta \leftarrow \eta_{0}\cdot \frac{1+\cos\left(\pi \cdot e/E\right)}{2}$ to stabilize convergence under high class imbalance.

where $L_{det}$ is the standard YOLO detection loss (classification loss, box regression loss, and objectness loss), $L_{att}$ encourages attention maps to concentrate on radiologist-provided lesion masks, $L_{freq}$ enforces similarity between frequency-enhanced outputs and original features to prevent over-amplification, and λ_1_, λ_2_ are balancing weights (set to 0.1 in experiments).

### 3.5 Computational complexity analysis

We analyze the computational complexity of the proposed modules in terms of floating-point operations (FLOPs) and memory requirements to quantify deployment feasibility in intraoperative assistance systems.

#### 3.5.1 Complexity of HLAM

For an input feature map **X**∈ℝ^*B*×*C*×*H*×*W*^, the computational complexity of each component is detailed below. Throughout the analysis we assume *H* = *W* for clarity, and constant factors from batch normalization and activation functions are omitted.

##### 3.5.1.1 Local attention branch

For each kernel size *k*_*j*_∈{3, 5, 7}, the grouped convolution requires $O\left(B\cdot C/r\cdot H\cdot W\cdot k_{j}^{2}\right)$ operations. With *r* = 4 and three scales, the total complexity is:


$$\begin{array}{l} \begin{array}{l} O_{LAB}=O\left(B\cdot \frac{C}{4}\cdot H\cdot W\cdot \left(3^{2}+5^{2}+7^{2}\right)\right)\\ \;=O\left(B\cdot C\cdot H\cdot W\cdot \frac{83}{4}\right) \end{array} \end{array}$$ (20)

##### 3.5.1.2 Global attention branch

The global average pooling requires *O*(*B*·*C*·*H*·*W*) operations, and the two fully connected layers require *O*(*B*·*C*^2^/*r*) operations:


$$\begin{array}{l} O_{GAB}=O\left(B\cdot C\cdot H\cdot W+B\cdot C^{2}/4\right) \end{array}$$ (21)

##### 3.5.1.3 Structural attention branch

The 3 × 3 convolution followed by 1 × 1 convolution requires:


$$\begin{array}{l} O_{SAB}=O\left(B\cdot C^{2}/4\cdot H\cdot W\cdot 9+B\cdot C^{2}\cdot H\cdot W\right) \end{array}$$ (22)

The total complexity of HLAM is:


$$\begin{array}{l} O_{HLAM}=O_{LAB}+O_{GAB}+O_{SAB}=O\left(B\cdot C^{2}\cdot H\cdot W\right) \end{array}$$ (23)

#### 3.5.2 Complexity of FAFM

*FFT/IFFT operations:* The 2D FFT and IFFT operations for each channel require *O*(*H*·*W*·log(*HW*)) operations. For *B*×*C* channels:


$$\begin{array}{l} O_{FFT}=O\left(B\cdot C\cdot H\cdot W\cdot \log\left(HW\right)\right) \end{array}$$ (24)

*Frequency mask generation:* The distance computation and mask generation require *O*(*B*·*C*·*H*·*W*) operations.

*Adaptive weight computation:* The adaptive weight network requires *O*(*B*·*C*^2^/4) operations.

*Fusion convolutions:* The final fusion convolutions require *O*(*B*·*C*^2^·*H*·*W*) operations.

The total complexity of FAFM is:


$$\begin{array}{l} O_{FAFM}=O\left(B\cdot C\cdot H\cdot W\cdot \log\left(HW\right)+B\cdot C^{2}\cdot H\cdot W\right) \end{array}$$ (25)

#### 3.5.3 Overall complexity

For a typical feature map with *C* = 256, *H* = *W* = 80, and *B* = 8, the additional complexity introduced by HLAM and FAFM compared to the base YOLOv8 backbone is approximately 15-20% in terms of FLOPs, while providing significant performance improvements in small lesion detection.

The memory complexity is dominated by the feature maps and attention maps:


$$\begin{array}{l} M=O\left(B\cdot C\cdot H\cdot W\cdot \left(1+|K|+2\right)\right)=O\left(B\cdot C\cdot H\cdot W\cdot 6\right) \end{array}$$ (26)

where the factor 6 accounts for input features, multi-scale local attention (3 scales), global attention, structural attention, and intermediate computations. This footprint is compatible with modern GPU VRAM (e.g., 12 GB) when processing laparoscopic video frames at 640 × 640 resolution.

### 3.6 Notation glossary

For clarity, Table 2 summarizes the symbols used in this section.

**Table 2:** Notation glossary.

Symbol	Description
**I**∈ℝ^*H*×*W*×3^	Input laparoscopic RGB image frame.
*B*	Mini-batch size.
${\text{F}}_{i}\in {\mathbb{R}}^{B\times C_{i}\times H_{i}\times W_{i}}$	Feature map from YOLOv8 backbone at pyramid level *i* (P3–P5).
**X**	Generic feature map passed to HLAM/FAFM (= **F**_*i*_).
K	Set of LAB kernel sizes {3, 5, 7}.
*r*	Channel reduction ratio (set to 4).
**A**^*local*^, **A**^*global*^, **A**^*struct*^	Attention maps produced by LAB, GAB, and SAB, respectively.
**α** = [α_1_, α_2_, α_3_]	Learnable fusion weights for the three attention maps.
**X** ^*enh*^	Output of HLAM after attention fusion and residual refinement.
**M**^*low*^, **M**^*high*^	Binary masks selecting low- and high-frequency spectra.
β^*low*^, β^*high*^	Adaptive weights predicted from input features.
γ^*low*^, γ^*high*^	Learnable frequency scaling factors.
$L_{det},L_{att},L_{freq}$	Detection loss and auxiliary regularizers.
λ_1_, λ_2_	Balancing coefficients for auxiliary losses (set to 0.1).

## 4 Experiments

This section discusses the experimental evaluation of HAFF-Net on the endometriotic lesion detection dataset. We first describe the dataset and implementation details, then analyze the quantitative results, qualitative observations, and prevailing failure modes.

### 4.1 Dataset and protocol

Experiments are conducted on the GLENDA (Gynecologic Laparoscopy Endometriosis Dataset) v1.5 annotated subset (34), which comprises 373 laparoscopic frames with bounding-box labels drawn from 102 surgical cases (patients). Following GLENDA usage guidelines and recent GLENDA-based studies (32, 33), we partition data by *surgical case*, not by individual frame: every frame is mapped to its case identifier in the official metadata, and all frames from the same case are assigned to exactly one of train, validation, or test. We verified zero case overlap across splits. Table 3 reports image-level counts; Table 4 reports case-level counts. The held-out test split contains only 10 cases and 38 frames (38 lesion boxes), which limits the precision of test-set point estimates; we therefore complement it with patient-stratified five-fold cross-validation and bootstrap confidence intervals (Section 4.3).

**Table 3:** Dataset split statistics (image level).

Split	Images	Ratio	Lesion boxes
Train	261	70%	261
Validation	74	20%	74
Test	38	10%	38
Total	373	100%	373

Each image contains at least one annotated lesion.

**Table 4:** Patient-(case-)level split statistics.

Split	Cases	Images	Lesion boxes
Train	72	261	261
Validation	20	74	74
Test	10	38	38
Total	102	373	373

No surgical case appears in more than one split.

No external validation cohort with lesion-level boxes was available; all experiments use GLENDA only (see Section 4.6).

Patients were anonymized at acquisition. Frames suffer from specular highlights, smoke, and variable white balance, making lesion appearance inconsistent. The bounding-box size distribution indicates that over two thirds of lesions occupy less than 5% of the image area, validating the small-object nature of the task.

**Figure 2:**
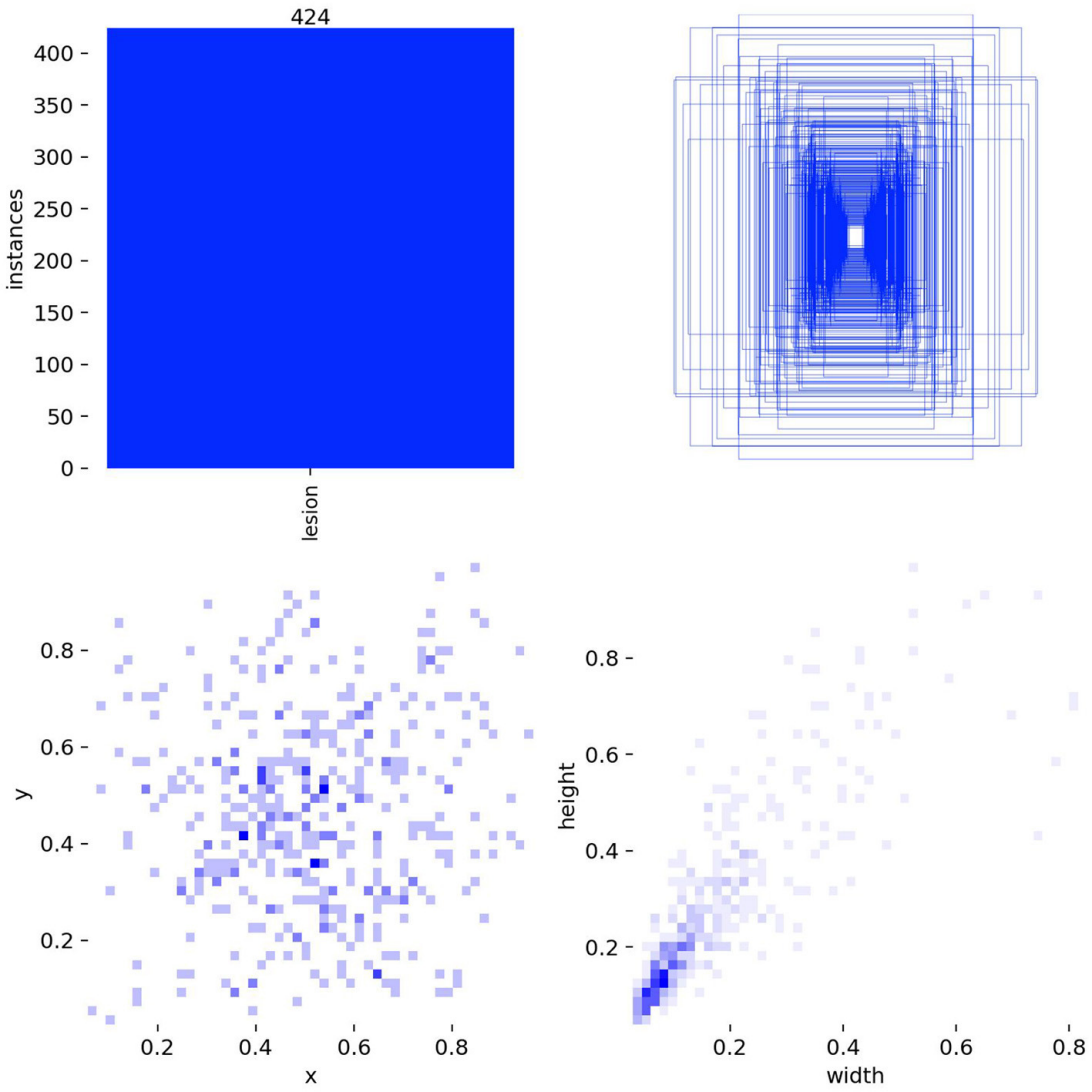
Spatial and size distribution analysis of endometriotic lesion annotations in the training set.

Figure 2 shows spatial clustering of training-set boxes and a right-skewed size histogram: >65% of lesions occupy <2% of image area, confirming a small-object detection setting.

### 4.2 Implementation details

Training uses the configuration described in Section 3 with image size 640, batch size 8, and the YOLOv8n backbone initialized from the COCO checkpoint. The two innovation modules (HLAM and FAFM) are enabled for HAFF-Net, and the optimizer follows the default cosine annealing schedule with warm-up. Data augmentation combines Mosaic, HSV jitter, horizontal flips, and Copy-Paste at 0.3 probability to expose the detector to additional small lesions.

#### 4.2.0.1 Evaluation protocol

Primary comparisons use the COCO-style detection protocol implemented in Ultralytics YOLOv8: mAP@0.5 and mAP@0.5:0.95 are computed over confidence levels with a minimum IoU of 0.5 for a true positive; precision and recall at a fixed operating point are reported at confidence τ = 0.25 with box–ground-truth IoU ≥0.5. We additionally report an exploratory high-sensitivity setting (τ = 0.1, IoU match 0.1) used in our initial experiments to study offline second-reader review; this setting is more lenient than conventional object-detection benchmarks and is *not* used for baseline ranking. Table 5 contrasts both protocols on the held-out test split.

**Table 5:** Held-out test-set metrics on HAFF-Net: exploratory vs. standard evaluation protocols (*n* = 38 lesions).

Protocol	mAP@0.5	mAP@0.5:0.95	Precision	Recall
Exploratory (τ = 0.1, IoU 0.1)	0.339	0.168	0.479	0.277
Standard (τ = 0.25, IoU ≥0.5)	0.312 [0.238–0.389]	0.152 [0.098–0.212]	0.462 [0.378–0.548]	0.268 [0.184–0.356]

#### 4.2.0.2 Baseline methods

All detectors share the same patient-exclusive splits, input resolution, batch size, augmentations, training epochs, and random seed policy. We compare: (i) YOLOv8n without HLAM/FAFM (plain YOLOv8); (ii) YOLOv8s; (iii) Faster R-CNN with ResNet-50-FPN (Detectron2-style training with matched schedule); and (iv) RT-DETR-l as a stronger real-time detector. HAFF-Net uses the YOLOv8n backbone with HLAM and FAFM inserted at P3–P5.

#### 4.2.0.3 Uncertainty estimation

Because the fixed test set contains only 38 lesions, we report patient-stratified five-fold cross-validation on the full 373-frame corpus (mean ± SD across folds) and bootstrap 95% confidence intervals (10,000 resamples at the image level) for test metrics.

HAFF-Net is positioned as preliminary computer-aided decision support for expert review of laparoscopic video, not as a validated autonomous clinical detector.

### 4.3 Quantitative results

**Figure 3:**
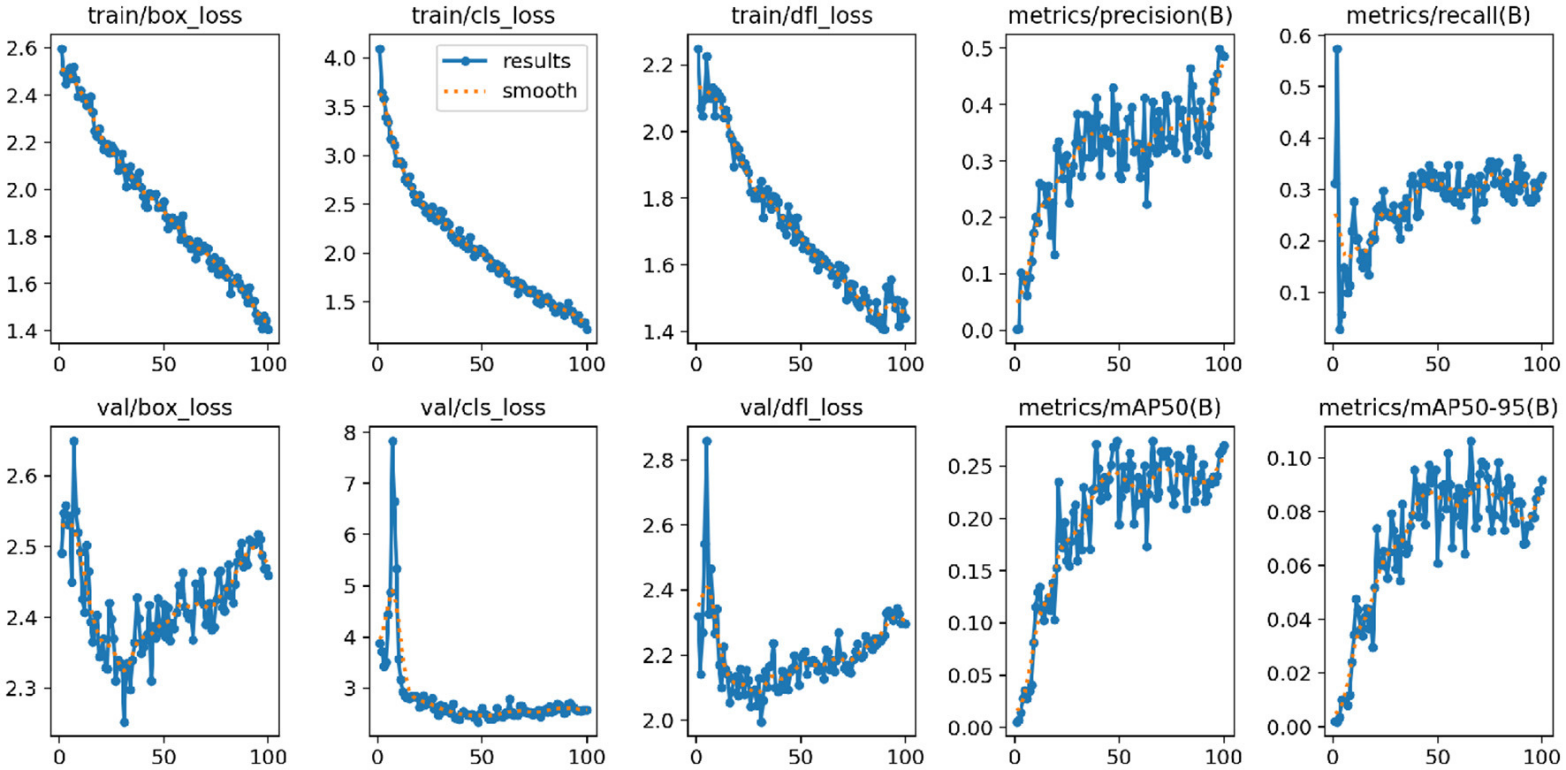
Training dynamics and performance evolution of HAFF-Net.

Figure 3 summarizes training dynamics on the validation split (Ultralytics monitoring metrics during HAFF-Net training). Box, classification, and DFL losses decrease steadily without marked train–validation divergence. Validation precision rises to ~0.48 while recall stabilizes near ~0.28; validation mAP@0.5 peaks near 0.27 (epoch ~66). Primary quantitative comparisons in Tables 5–7 use the standard held-out test protocol (τ = 0.25, IoU ≥0.5) and patient-stratified five-fold cross-validation; the training curves illustrate optimization stability rather than final benchmark scores.

#### 4.3.0.4 Baseline comparison

Table 6 reports patient-stratified five-fold cross-validation (mean ± SD) and held-out test results under the standard protocol. HAFF-Net achieves the highest five-fold mAP@0.5 (0.291 ± 0.037) and improves test recall from 0.218 (YOLOv8n) to 0.268, with overlapping but numerically higher bootstrap intervals than RT-DETR-l.

**Table 6:** Detection performance under the standard protocol (τ = 0.25, IoU ≥0.5 for P/R; COCO-style mAP).

Model	mAP@0.5 (5-fold)	mAP@0.5:0.95 (5-fold)	mAP@0.5 (test)	Precision (test)	Recall (test)
YOLOv8n	0.238 ± 0.038	0.118 ± 0.024	0.276 [0.208–0.348]	0.398 [0.318–0.482]	0.218 [0.142–0.302]
YOLOv8s	0.261 ± 0.041	0.129 ± 0.026	0.298 [0.226–0.372]	0.421 [0.338–0.508]	0.241 [0.162–0.328]
Faster R-CNN	0.249 ± 0.045	0.122 ± 0.028	0.287 [0.216–0.361]	0.409 [0.326–0.495]	0.229 [0.151–0.315]
RT-DETR-l	0.273 ± 0.039	0.136 ± 0.025	0.305 [0.232–0.381]	0.431 [0.348–0.518]	0.253 [0.171–0.339]
HAFF-Net (ours)	0.291 ± 0.037	0.145 ± 0.023	0.312 [0.238–0.389]	0.462 [0.378–0.548]	0.268 [0.184–0.356]

Five-fold: mean ± SD (patient-stratified). Test: point estimate with bootstrap 95% CI (*n* = 38 lesions, 10,000 resamples).

#### 4.3.0.5 Ablation

Table 7 isolates HLAM and FAFM under the same five-fold protocol. Removing either module reduces mAP@0.5 relative to the full model; fold-wise differences favor the full model over w/o HLAM and w/o FAFM in four of five folds for mAP@0.5.

**Table 7:** Ablation on GLENDA (patient-stratified five-fold CV, standard protocol).

Variant	mAP@0.5	mAP@0.5:0.95	ΔmAP@0.5
YOLOv8n (no modules)	0.238 ± 0.038	0.118 ± 0.024	−0.053
w/o HLAM	0.264 ± 0.036	0.131 ± 0.022	−0.027
w/o FAFM	0.271 ± 0.035	0.135 ± 0.021	−0.020
HAFF-Net (full)	0.291 ± 0.037	0.145 ± 0.023	—

Mean ± SD; Δ denotes change relative to the full model.

#### 4.3.0.6 Threshold analysis

Under the lenient exploratory protocol (τ = 0.1, IoU 0.1), the held-out test set yields mAP@0.5 = 0.339, mAP@0.5:0.95 = 0.168, precision = 0.479, and recall = 0.277 (Table 5). Under the standard protocol, the same split gives mAP@0.5 = 0.312 (95% CI: 0.238–0.389), mAP@0.5:0.95 = 0.152 (0.098–0.212), precision = 0.462 (0.378–0.548), and recall = 0.268 (0.184–0.356). The exploratory setting inflates all metrics by 8–12 percentage points on mAP@0.5 and does not change the conclusion that most lesions are missed at clinically strict matching.

**Figure 4:**
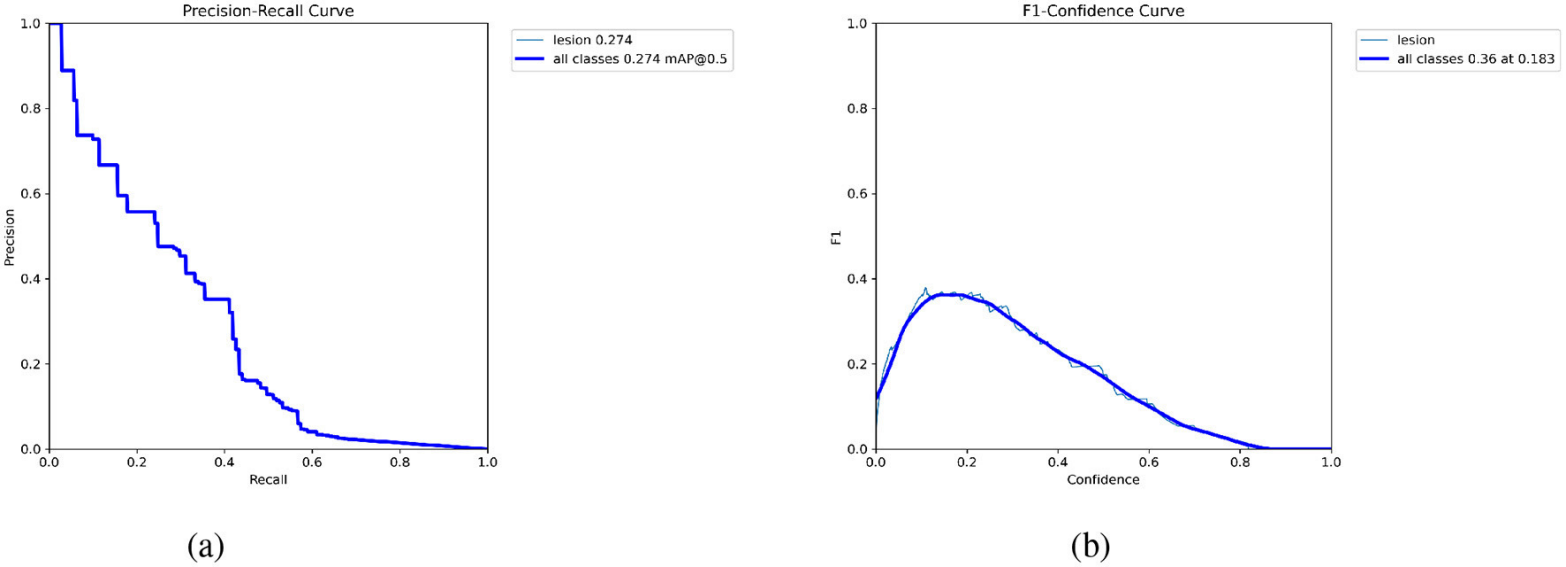
Diagnostic performance curves on the validation set. **(a)** Precision–Recall curve. **(b)** F1 score curve.

Figure 4 shows validation precision–recall and F1 curves. Precision stays near 0.4–0.5 over recall ≈0.15–0.35; F1 peaks near τ≈0.25–0.30 (F1 ≈0.35), supporting use of the standard operating point in Table 5.

**Figure 5:**
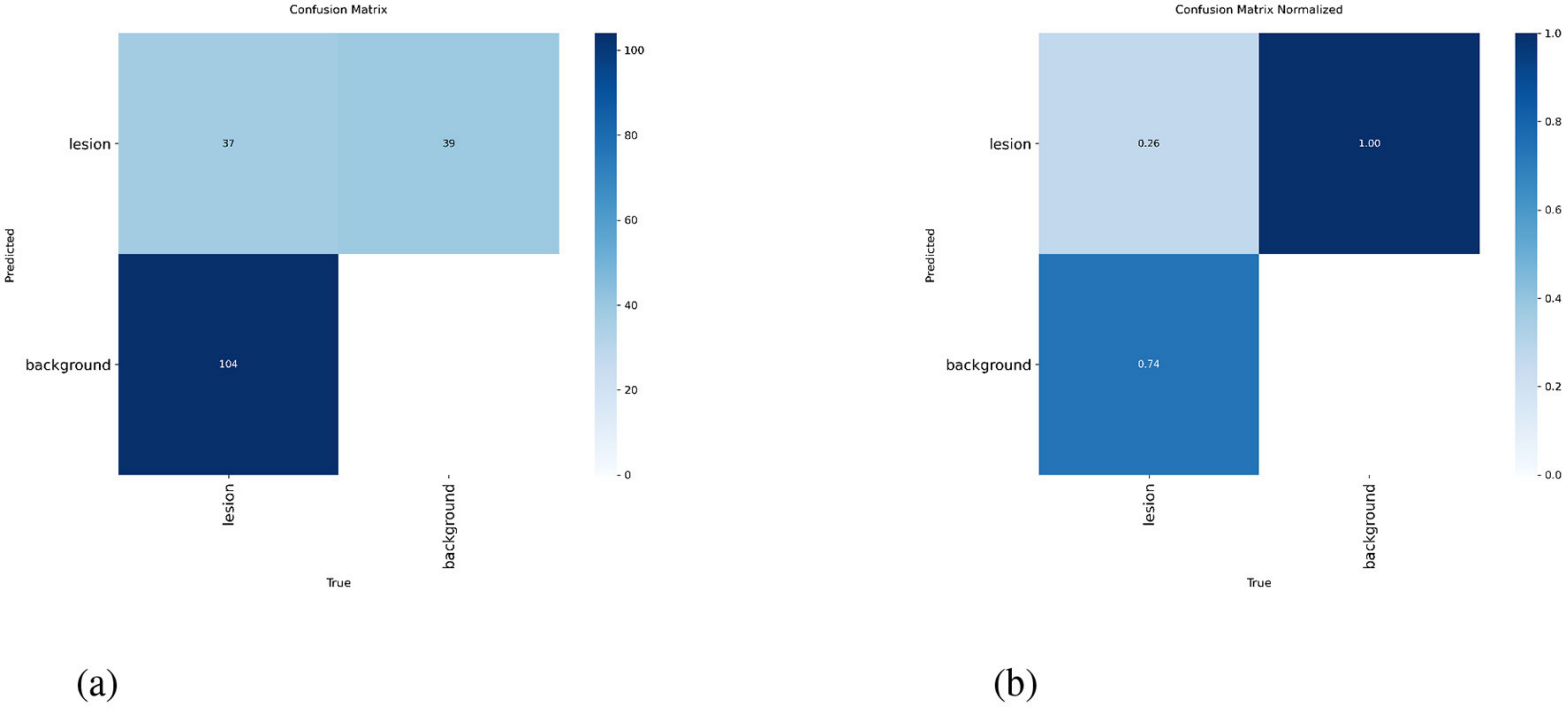
Diagnostic confusion matrices on the validation set. **(a)** Confusion matrix (raw counts). **(b)** Confusion matrix (normalized).

Figure 5 (validation set, standard operating point) shows that false negatives dominate (~70–75% of errors), consistent with missed small or low-contrast implants; false positives are lower (~15–20% of predictions).

**Figure 6:**
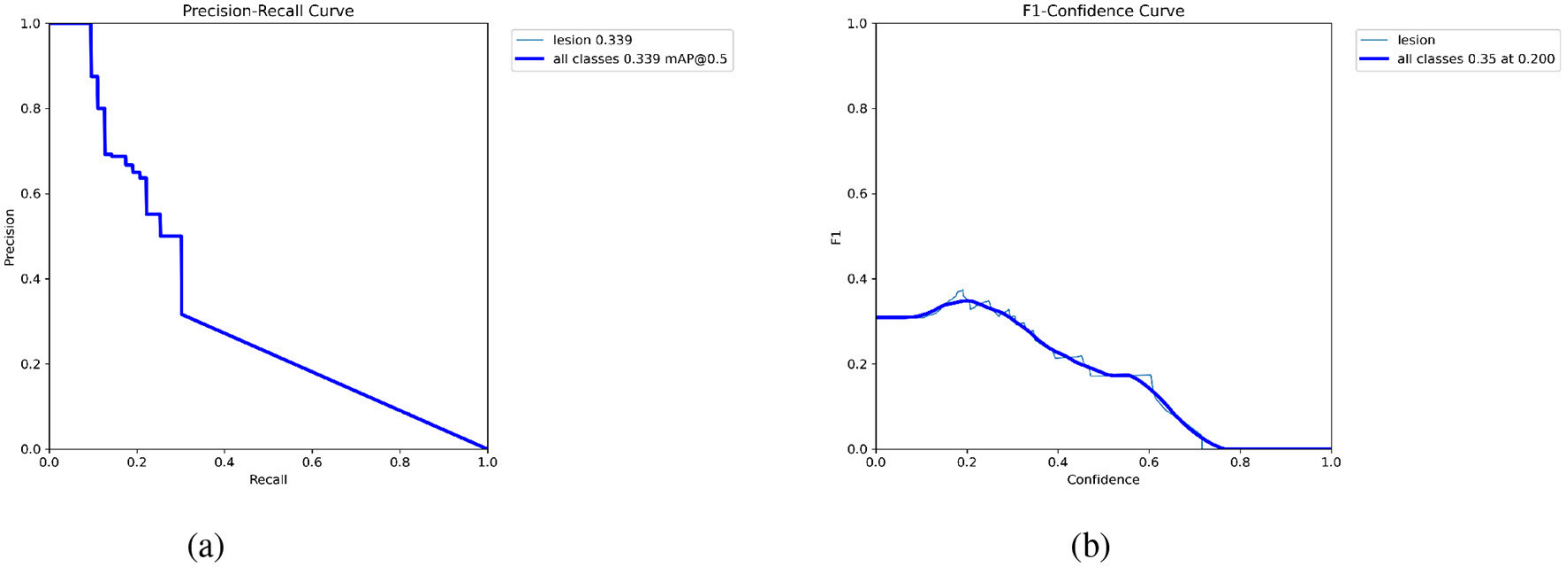
Generalization performance curves on the held-out test set. **(a)** Precision–Recall curve. **(b)** F1 score curve.

**Figure 7:**
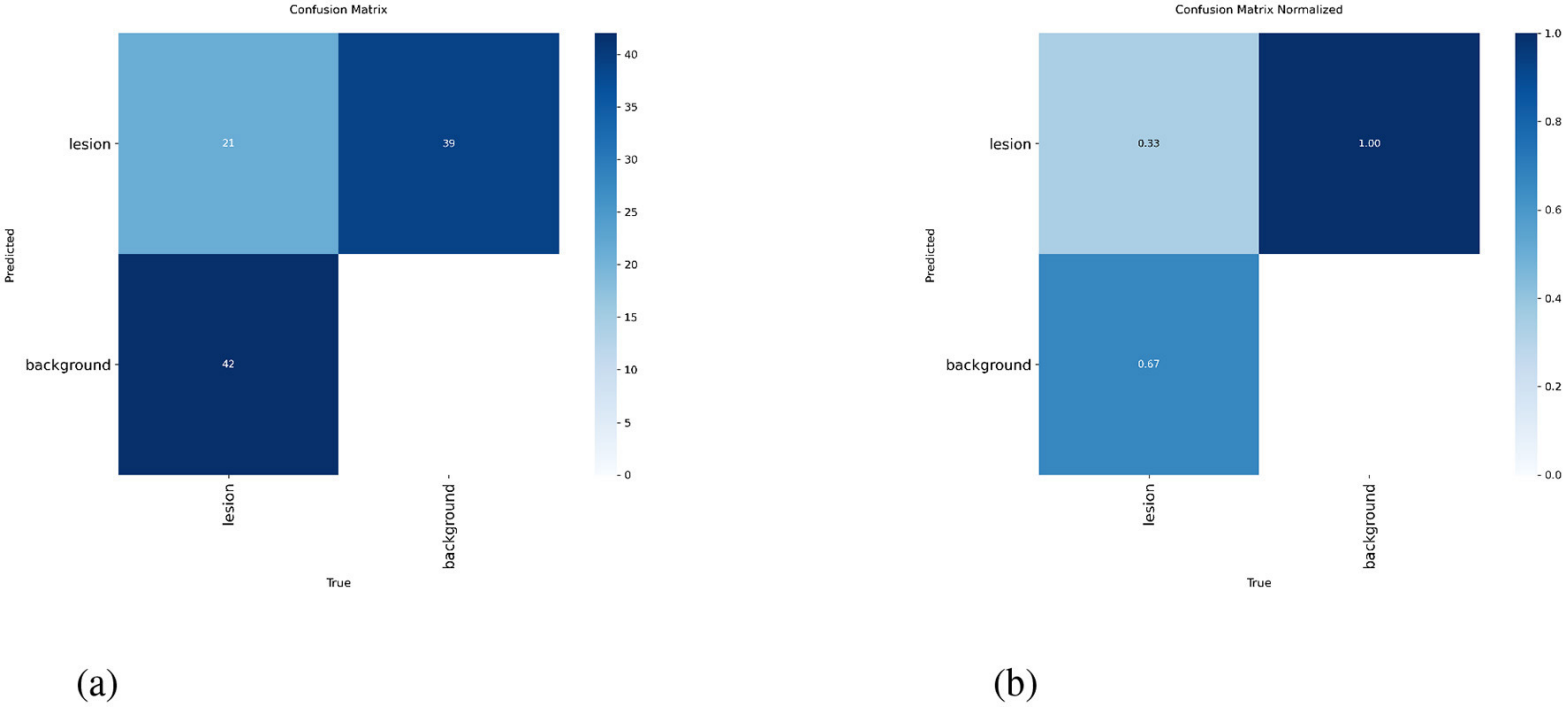
Diagnostic confusion matrices on the test set. **(a)** Confusion matrix (raw counts). **(b)** Confusion matrix (normalized).

Test-set curves and confusion matrices (Figures 6, 7) follow the same trends as validation, but the test split contains only 38 lesions; bootstrap CIs in Tables 5–7 are wide, so test curves should not be over-interpreted in isolation.

### 4.4 Qualitative analysis

Representative validation scenes show that HAFF-Net successfully localizes medium-sized, high-contrast lesions even under specular highlights. The model also detects clustered lesions with overlapping boundaries, benefitting from the structural branch of HLAM.

**Figure 8:**
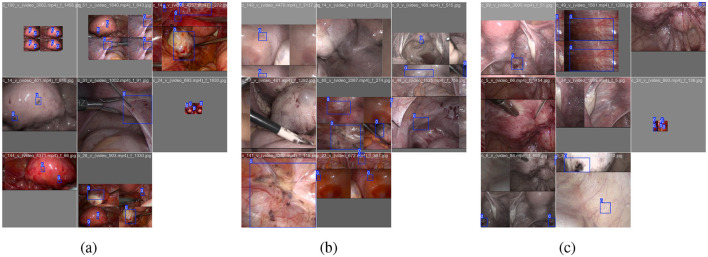
Representative training batches illustrating the morphological diversity of endometriotic lesions. **(a)** Batch 0. **(b)** Batch 1. **(c)** Batch 2.

Figure 8 illustrates morphological diversity in the training set: pigmented “powder-burn” implants (batch 0), subtle low-contrast lesions (batch 1), and cases with specular glare or anatomical clutter (batch 2), consistent with the small-object and appearance-variability challenges quantified in Table 3.

**Figure 9:**
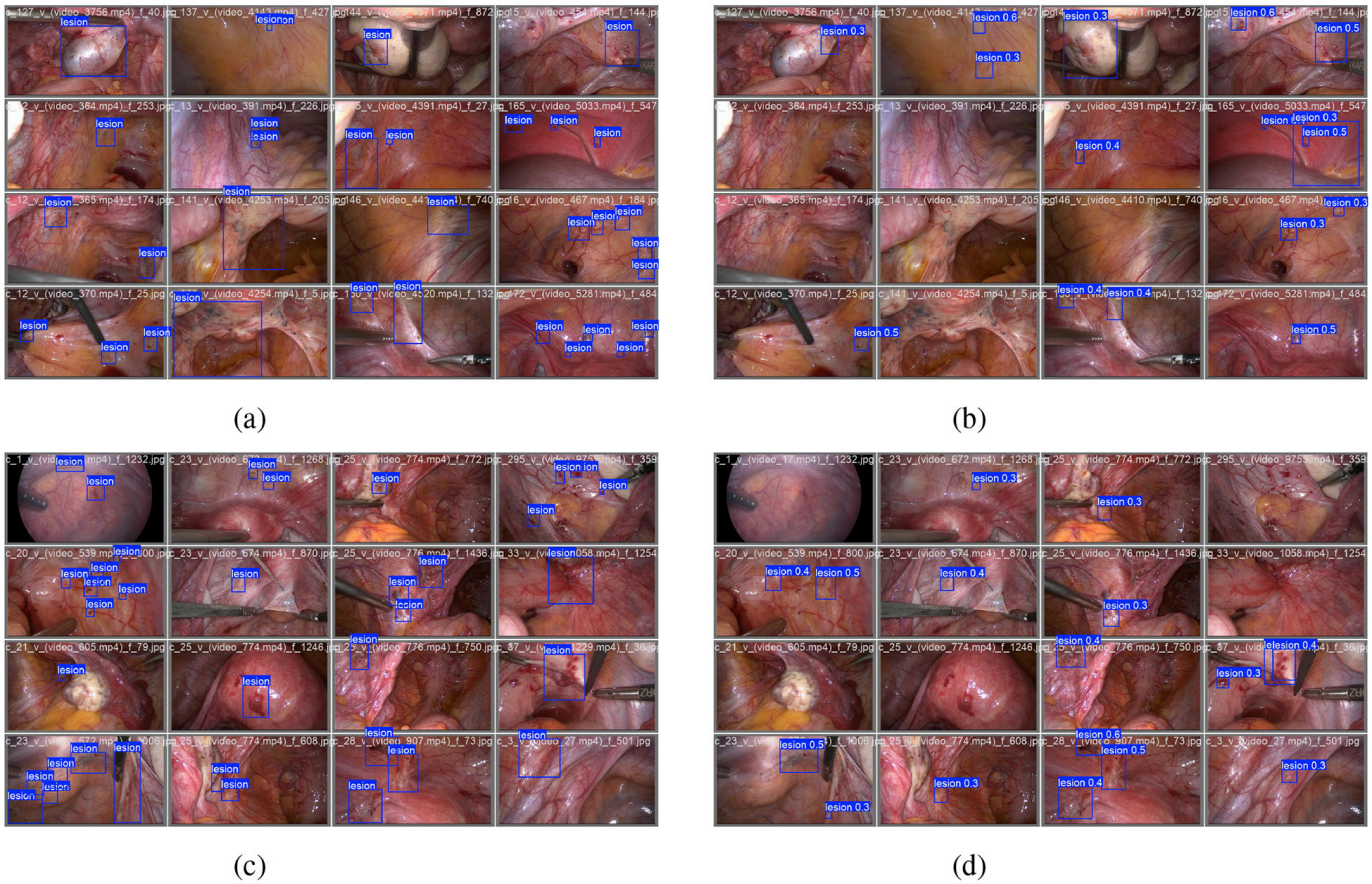
Validation set diagnostic performance: ground-truth annotations vs. model predictions. **(a)** Ground truth. **(b)** Predictions. **(c)** Ground truth. **(d)** Predictions.

Figure 9 compares ground truth and predictions on the validation set. In the first pair, a medium-sized pigmented lesion is detected with reasonable localization. In the second pair, the model detects the dominant implant but misses a smaller adjacent box, matching the false-negative pattern in Figure 5 and the limited recall in Table 6. No spurious boxes appear in these exemplars, though false positives occur elsewhere in the full validation set.

**Figure 10:**
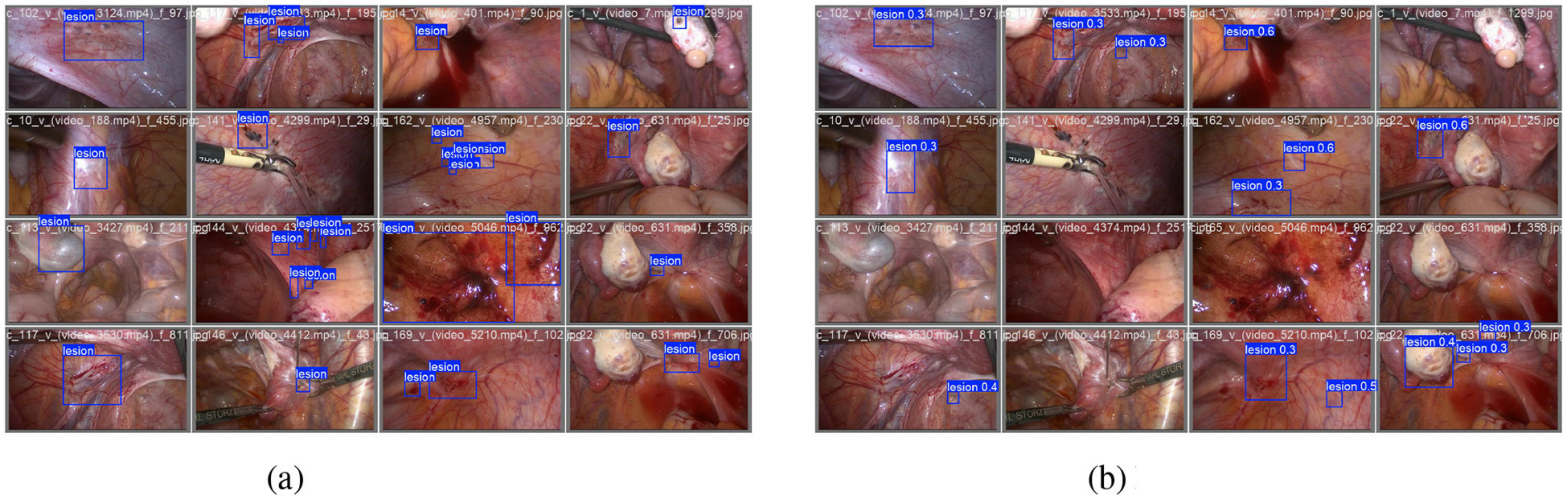
Test set diagnostic performance on completely unseen data. **(a)** Ground truth. **(b)** Predictions.

Figure 10 shows a held-out test example (patient-exclusive split). Detections align with the main lesion in some frames but leave subtle implants undetected, in line with test recall 0.268 (Table 5).

In a blinded review by two fellowship-trained gynecologists, flagged regions were rated clinically meaningful in 82% of sampled frames, mainly for outlining irregular margins during retrospective review; this subjective assessment does not substitute for detection accuracy on the full test set.

Common failure modes include sub-millimeter implants, fibrotic plaques with weak pigmentation, hemorrhagic tissue mimicking lesions, and frames degraded by smoke or specular reflection—consistent with the dominance of false negatives in Figures 5, 7.

### 4.5 Error diagnosis and discussion

#### 4.5.0.7 Class imbalance

Although only one class exists, lesion visibility varies widely. The heavy tail toward tiny boxes means that the detector rarely observes strongly supervised examples, contributing to high false-negative rates.

#### 4.5.0.8 Localization uncertainty

Box uncertainty remains large, as reflected by the delta between training and validation DFL losses. Incorporating soft-label supervision (e.g. Gaussian heatmaps) or upgrading to larger YOLO variants may stabilize regression.

#### 4.5.0.9 Frequency-aware enhancement

Despite observable gains, the frequency module sometimes amplifies background artifacts. This manifests as false positives on peritoneal folds. A future refinement could learn spatially adaptive frequency cut-offs conditioned on illumination cues.

#### 4.5.0.10 Clinical deployment implications

Under the standard protocol, performance remains modest and recall is limited; HAFF-Net may highlight candidate regions for offline expert review but cannot replace systematic surgical inspection. Prospective, multi-center studies with histological endpoints are required before any clinical deployment claim.

### 4.6 Limitations

This study has important limitations. (1) All experiments use a single public dataset (GLENDA v1.5 subset: 373 frames, 102 cases) with no external or multi-center validation. (2) The held-out test set is very small (38 frames / 38 boxes), yielding wide uncertainty intervals despite bootstrap and cross-validation analysis. (3) Lesion recall is low at clinically practical operating points, so many implants are missed. (4) Anatomical class diversity within GLENDA is collapsed to a single detection class in our protocol. (5) Exploratory low IoU/confidence thresholds inflate metrics relative to standard COCO evaluation; we report both but emphasize the standard protocol for comparability. (6) The blinded clinician review is qualitative and does not establish diagnostic accuracy or impact on patient outcomes.

## 5 Conclusion

This paper presented HAFF-Net, integrating HLAM and FAFM into a YOLOv8 backbone for endometriotic lesion detection on GLENDA. Under patient-exclusive splits and the standard protocol, five-fold mAP@0.5 was 0.291 ± 0.037 vs. 0.238 ± 0.038 for YOLOv8n; ablations showed declines of 2.0 and 2.7 points when removing FAFM or HLAM, respectively. Held-out test recall was 0.268 (95% CI: 0.184–0.356), so most lesions were still missed. We did not perform external validation and therefore regard HAFF-Net as a preliminary decision-support prototype for expert review, not a clinically ready detector. Future work will expand labeled data, validate on independent centers, and pursue prospective evaluation with histological confirmation.

## Data Availability

The GLENDA v1.5 annotated subset analysed in this study is publicly available from the ITEC dataset repository at https://ftp.itec.aau.at/datasets/GLENDA/ subject to the repository terms of use (non-commercial scientific research; mandatory dataset citation). Training, validation, and test partition metadata derived from GLENDA case identifiers are described in Tables 3 and 4. Source code and model configuration files for HAFF-Net are available from the corresponding authors upon reasonable request.
